# A Comparison Between Histology and Rapid Urease Test in the Diagnosis of Helicobacter Pylori in Gastric Biopsies: A Systematic Review

**DOI:** 10.7759/cureus.39360

**Published:** 2023-05-22

**Authors:** Neway A Urgessa, Prabhitha Geethakumari, Prathima Kampa, Rakesh Parchuri, Renu Bhandari, Ali R Alnasser, Aqsa Akram, Saikat Kar, Fatema Osman, Ghadi D Mashat, Hadrian Hoang-Vu Tran, Ana P Arcia Franchini

**Affiliations:** 1 Pathology, California Institute of Behavioral Neurosciences & Psychology, Fairfield, USA; 2 Internal Medicine, California Institute of Behavioral Neurosciences & Psychology, Fairfield, USA; 3 Internal Medicine/Family Medicine, California Institute of Behavioral Neurosciences & Psychology, Fairfield, USA; 4 Internal Medicine, Manipal College of Medical Sciences, Kaski, NPL; 5 General Surgery, California Institute of Behavioral Neurosciences & Psychology, Fairfield, USA; 6 Internal Medicine, Dallah Hospital, Riyadh, SAU; 7 Neurosciences and Psychology, California Institute of Behavioral Neurosciences & Psychology, Fairfield, USA; 8 Pediatrics, California Institute of Behavioral Neurosciences & Psychology, Fairfield, USA; 9 Research, California Institute of Behavioral Neurosciences & Psychology, Fairfield, USA

**Keywords:** peptic ulcer, urease test, histology, diagnostic, invasive, helicobacter pylori

## Abstract

*Helicobacter pylori (H. pylori)* is a gram-negative aerobic pathogen that primarily colonizes the gastric mucosa. Peptic ulcer disease, atrophic gastritis, gastric cancer, and mucosal-associated lymphoid tissue lymphoma have all been linked to chronic *H. pylori *infection. Hence, it is critical to diagnose and treat it as early as possible. There are both invasive and noninvasive tests available to detect it. In this review, the diagnostic abilities of two invasive tests - histology and the rapid urease test (RUT) - are compared in a variety of clinical situations.

This systematic review was carried out using the Preferred Reporting Items for Systematic Reviews and Meta-analyses (PRISMA) 2020 checklist. We performed a literature search using the PubMed and Google Scholar databases in accordance with the eligibility criteria and ultimately selected eight articles for final analysis. The Newcastle-Ottawa scale adapted for cross-sectional studies, the Scale for the Assessment of Narrative Review Articles (SANRA), and the PRISMA 2020 checklist were used to assess the quality of selected articles for cross-sectional studies, traditional literature reviews, and systematic reviews, respectively. According to the findings of the review, both histology and the RUT have high sensitivity and specificity in diagnosing *H. pylori *though this varies depending on the clinical situation, making one test superior to the other. Neither of these tests can be considered the gold standard method on its own. Hence, using at least two diagnostic tests at the same time is critical for ensuring high sensitivity and specificity while accurately diagnosing the pathogen.

## Introduction and background

*Helicobacter pylori (H. pylori)* is an aerobic, spiral-shaped gram-negative bacteria that live on the mucosa of the stomach [1-4]. It was cultured for the first time in 1983 and initially reported as urease-negative bacteria. However, when other researchers tried to replicate this finding, they correctly identified it as urease-positive [5]. *H. pylori* infection is very common worldwide and has a prevalence rate of 80-90% in developing countries and 40-50% in developed countries [6-8]. In the United States (US), the infection rate is close to 30-40% [5]. *H. pylori *is mostly transmitted via fecal-oral and oral-oral routes [9,10]. It produces the enzyme urease, which changes urea in the stomach to ammonia and carbon dioxide (CO_2_). The ammonia changes the acidic stomach PH to alkali, making it suitable for the bacteria to live in. It also produces exotoxins such as cytotoxin-associated gene A (Cag A) and vacuolating cytotoxin A (Vac A), which disrupt the integrity of the stomach mucosa, thereby causing inflammation and ulceration [9].

*H. pylori* has been linked to the occurrence of vitamin B_12_ deficiency, atrophic gastritis, gastric and duodenal ulcers, gastric cancer, and mucosal-associated lymphoid tissue (MALT) lymphoma [2,5,8,10]. As a result, the World Health Organization (WHO) labeled it a "definite biological carcinogen" in 1994 [2,7,11]. It is important to diagnose *H. pylori *infection accurately for the proper management and follow-up of patients. Currently, a single gold standard diagnostic test is not available. The type of test to be used varies depending on the clinical situation, the probability of infection, the cost of the test, and its availability [2,12,13]. For diagnosis and monitoring, both invasive and noninvasive tests are used. Histology with special stains, rapid urease tests (RUT), culture, and polymerase chain reaction (PCR) are examples of invasive tests. These tests require an endoscopy and a biopsy. Serology, stool antigen testing, and urea breath testing are examples of noninvasive tests [1,2,10]. This review compares the efficacy of two invasive tests - histology and RUT- in the detection of *H. pylori* in various circumstances.

## Review

Methods

This systematic review was performed using the Preferred Reporting Items for Systematic Reviews and Meta-analyses (PRISMA) 2020 checklist [14].

Search Strategy

We carried out a systematic search of the literature, published between January 2012 and June 2022, based on our eligibility criteria by using the databases PubMed and Google Scholar. The keywords and medical subject heading (MeSH) strategies we used are shown in Table 1.

**Table 1:** Keywords and MeSH terms used in the search MeSH: Medical Subject Headings; *H. pylori: Helicobacter pylori*

Key terms	Database	
*Helicobacter pylori *AND invasive diagnostic test	PubMed	
*Helicobacter pylori *diagnosis AND peptic ulcer	PubMed	
*Helicobacter pylori* AND urease test	PubMed	
*Helicobacter pylori *AND histologic diagnosis	PubMed	
*Helicobacter pylori *AND histology AND urease test	PubMed	Google Scholar
MeSH terms		
(Helicobacter pylori OR H. pylori ( "Helicobacter pylori/analysis"[Majr] OR "Helicobacter pylori/microbiology"[Majr] OR "Helicobacter pylori/pathogenicity"[Majr] )) AND (Urease ( "Urease/analysis"[Majr] OR "Urease/anatomy and histology"[Majr] OR "Urease/cytology"[Majr] OR "Urease/etiology"[Majr] OR "Urease/immunology"[Majr]))	PubMed	
(Helicobacter pylori OR H. pylori ( "Helicobacter pylori/analysis"[Majr] OR "Helicobacter pylori/microbiology"[Majr] OR "Helicobacter pylori/pathogenicity"[Majr] )) AND (Peptic ulcer disease OR gastric ulcer OR duodenal ulcer OR stomach ulcer ( "Peptic Ulcer/analysis"[Majr] OR "Peptic Ulcer/cytology"[Majr] OR "Peptic Ulcer/diagnosis"[Majr] OR "Peptic Ulcer/immunology"[Majr] OR "Peptic Ulcer/microbiology"[Majr] OR "Peptic Ulcer/pathology"[Majr]))	PubMed	

Inclusion and Exclusion Criteria

Our inclusion and exclusion criteria are presented in Table 2.

**Table 2:** Inclusion and exclusion criteria

Inclusion criteria	Exclusion criteria
Studies published between 2012 and 2022	Studies outside mentioned time frame
Patients with dyspeptic symptoms	Patients without symptoms
Free full articles	Articles without free access
Only human studies	Animal studies
Article written in the English language	Articles written in other languages
Peer-reviewed articles	Articles not peer-reviewed
High-quality studies with a score >7	Low-quality studies with a score <7

Quality Assessment Tools

The quality assessment was performed independently by two reviewers. The tools we used are Newcastle-Ottawa Scale adapted for cross-sectional studies [15], the Scale for the Assessment of Narrative Review Article (SANRA) [16], and the PRISMA 2020 checklist [14] for cross-sectional studies, traditional literature reviews, and systematic reviews respectively. Each of these appraisal tools has specific criteria to evaluate the studies using a point system. A third reviewer was roped in to mitigate any discrepancies in the process of appraisal through discussion. Accordingly, only those articles that have high quality with a score >7 were selected for data extraction.

Data Extraction

The data were extracted using standardized recording tools by two reviewers independently. After we assessed the final number of articles as per the eligibility criteria mentioned above, the content of the selected data was searched for relevant information related to our research topic. Moreover, the information was divided into different subheadings to address the research question in the discussion section.

Results

Literature Search

**Figure 1:**
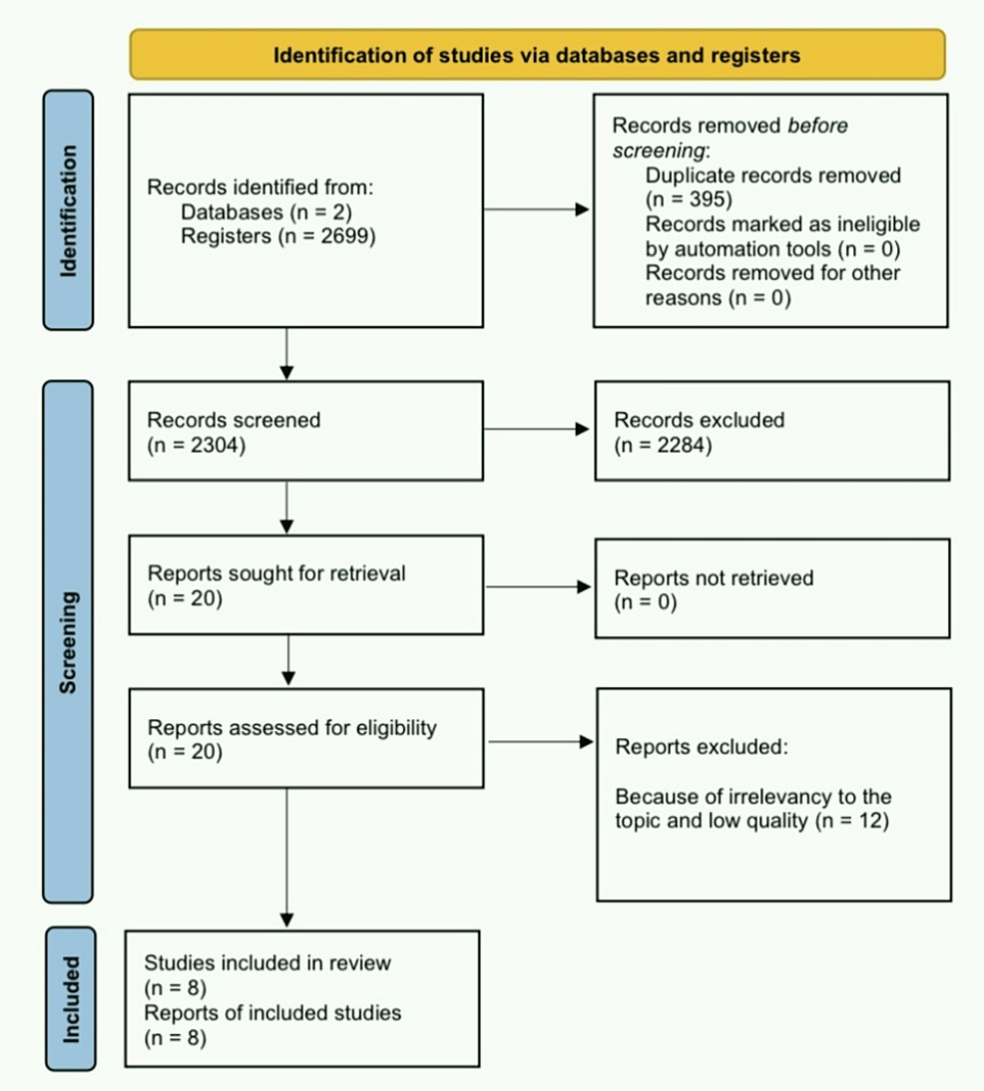
PRISMA flow chart PRISMA: Preferred Reporting Items for Systematic Reviews and Meta-analyses

After searching PubMed and Google Scholar using the search strategies mentioned above, we ended up with 2699 articles initially, of which 395 were duplicates. By going through the titles, 2215 articles were excluded, leaving only 89 articles, of which 69 were removed after reading through the abstract. We reviewed in detail the remaining 20 articles for relevance according to our topic and removed eight of them. Lastly, we selected eight articles for final review after excluding four articles by quality assessment. Figure 1 below is a PRISMA flow chart [14] demonstrating the search process and study selection.

Study Characteristics

Table 3 below summarizes the details in terms of authors, country of publication, and study design of the included studies. 

**Table 3:** Summary of the included articles - 1

Author	Country	Journal	Study design
Khalifehgholi et al., 2013 [1]	Iran	Iranian Journal of Microbiology	Cross-sectional study
Uotani and Graham, 2015 [5]	USA	Annals of Translational Medicine	Literature review
Karthikeyan and Sundaravadanan, 2021 [9]	India	Journal of Pharmaceutical Research International	Cross-sectional study
Lee et al., 2013 [11]	Taiwan	Gastroenterology Research and Practice	Cross-sectional study
Lee and Kim, 2015 [17]	South Korea	Annals of Translational Medicine	Literature review
Pourakbari et al., 2013 [18]	Iran	Brazilian Journal of Microbiology	Cross-sectional study
Seo et al., 2015 [19]	South Korea	World Journal of Clinical Pediatrics	Literature review
Vörhendi et al., 2020 [20]	Hungary	Therapeutic Advances in Gastroenterology	Systematic review

Table 4 below summarizes the details with regard to sample size where applicable, study quality, and conclusions of the included studies.

**Table 4:** Summary of the included articles - 2 RUT: rapid urease test; H. pylori: Helicobacter pylori; PCR: polymerase chain reaction; NA: not applicable

Author	Sample size	Quality of study (score)	Conclusion
Khalifehgholi et al., 2013 [1]	91	High (8)	Simultaneous use of biopsy-based tests is recommended
Uotani and Graham, 2015 [5]	NA	High (9)	RUT is best for screening tests of H. pylori and is not a gold standard test
Karthikeyan and Sundaravadanan, 2021 [9]	100	High (8)	Although histology and RUT have the same level of accuracy in detecting H. pylori, RUT may be a better choice in resource-constrained settings
Lee et al., 2013 [11]	246	High (9)	Antral biopsy histology is more accurate to diagnose H. pylori
Lee and Kim, 2015 [17]	NA	High (10)	Histology is an excellent method for detecting H. pylori and providing additional information about the gastric mucosa
Pourakbari et al., 2013 [18]	89	High (8)	Both histology and RUT are as accurate as PCR of biopsy in detecting H. pylori but are more time-consuming to perform
Seo et al., 2015 [19]	NA	High (9)	RUT has a lower sensitivity in diagnosing H. pylori in children under five since they have a low density as well as patchy distribution of the bacteria
Vörhendi et al., 2020 [20]	NA	High	Combined testing improves the sensitivity and specificity of H. pylori detection in peptic ulcer bleeding

Risk of Bias

The risk of bias in the studies selected was very low because we only included articles with high scores as determined by standard quality assessment tools [14-16]. The selected studies provide relevant information about the review topic.

Discussion

*H. pylori* is a gram-negative bacterium that has a preference for colonizing the gastric mucosa. It induces mucosal inflammation as well as injury ranging from asymptomatic gastritis to severe peptic ulcer disease and malignant lesions [2,10,12]. It is critical to treat it this condition immediately after diagnosis as it is always contagious and has an unpredictable progression. Diagnostic methods can be invasive or noninvasive. Each of these tests has advantages and disadvantages that make them useful in a variety of clinical situations [2,5].

Histology involves the direct examination of bacteria under a microscope after a biopsy has been performed and stained with hematoxylin and eosin (H&E) [2,8,17]. To increase the specificity of the test, special stains such as modified Giemsa stain, Warthin-Starry Silver stain, and immunohistochemical (IHC) stain can be used [17,21]. RUT, on the other hand, detects *H. pylori* through the existence or absence of a urease enzyme on the gastric mucosa. Urease activity is measured after a specimen of gastric tissue or mucosa is added to a urea-containing tube. If *H. pylori *is present in the sample, it will be hydrolyzed into CO_2_ and ammonia. Ammonia increases the PH of phenol red and changes its color from yellow to red. Furthermore, RUT detects only active infections, which makes it superior to serology [2,5,22].

Sensitivity, Specificity, Positive and Negative Predictive Value (PPV and NPV) of Histology and RUT

Histology with H&E stain has been confirmed to have a sensitivity of 69-93% and specificity of 87-90% [17]. RUT, on the other hand, has a sensitivity of about 80-100% and a specificity of 97-99% [5]. In one study conducted by Karthikeyan and Sundaravadanan [9], sensitivity, specificity, PPV, and NPV of RUT were reported as 95.24%, 89.19%, 93.79%, and 91.67%, respectively, when comparing histology and RUT by making histology the gold standard test. Another study done by Pourakbari et al. [18], making PCR the gold standard test, compared the two invasive tests. Accordingly, the study found that RUT has a sensitivity and specificity of 95.9% and 85% as compared to histology, which had 100% and 90% respectively. It also reported the PPV and NPV of RUT as 88.7% and 94.4%, in contrast to histology, which had a PPV of 92.5% and an NPV of 100%. Both studies concluded that the two invasive tests are comparable in terms of diagnosing *H. pylori* in dyspeptic patients.

The Effect of Site and Number of Biopsies on Sensitivity and Specificity of Both Tests

The updated Sydney system recommends that "for proper diagnosis of gastritis and *H. pylori* status, biopsy should be taken from five different sites. The locations are the lesser and greater curvature of the antrum, within 2-3 centimeters from the pylorus; the lesser curvature of the corpus about 4 centimeters proximal to the angulus; the middle portion of the greater curvature of the corpus approximately 8 centimeters from the cardia; and incisura angularis" [17]. This is because the greater the number of biopsy specimens, the fewer false negative results from sampling errors, and inadequate bacterial load or distribution, which ultimately increases the sensitivity and specificity of the tests [2,10,17,22].

A study conducted by Lee et al. [11], comparing invasive tests from different biopsy sites and making culture the gold standard test, reported the following results as shown in Table 5.

**Table 5:** Sensitivity, specificity, PPV, NPV, and accuracy of culture, histology, and RUT at different biopsy sites Adapted from [11]. Published under creative commons license. PPV: positive predictive value; NPV: negative predictive value; RUT: rapid urease test

Test	Sensitivity (%)	Specificity (%)	PPV (%)	NPV (%)	Accuracy (%)
Culture	91.46	100	100	95.91	97.15
Histology (antrum)	95.12	95.12	90.70	97.50	95.12
Histology (corpus)	76.83	96.95	92.65	89.33	90.24
Histology (antrum and corpus)	95.12	95.12	90.70	97.50	95.12
RUT (antrum)	64.63	100	100	84.97	88.21
RUT (corpus)	69.51	100	100	96.77	89.83
RUT (antrum and corpus)	86.59	100	100	93.71	95.52

In this study, histology from the corpus had the lowest sensitivity but histology from the antrum alone and that combined with the corpus had the highest sensitivity. In addition, RUT from antrum and corpus separately had the lowest sensitivity but the combined one had better sensitivity. Hence, this finding shows that to raise the sensitivity and lower false negativity, it is crucial to increase the number of biopsies and to take it from the proper sites.

Advantages and Limitations of Histology

Histology is one of the best methods for diagnosing *H. pylori* due to its high sensitivity and specificity. It also provides information on the degree of inflammation and associated pathology, including atrophic gastritis and intestinal metaplasia [13,21,23]. However, it has several limitations, such as higher costs, longer processing times, and reliance on the pathologist's skills [17,23].

Khalifehgholi et al. [1] conducted a study comparing five *H. pylori* diagnostic tests with samples taken from the antrum and corpus, making histology and PCR gold standard tests. This study found that histology had a sensitivity and specificity of 95.6% and 77.8%, whereas RUT had 95.6% and 100% respectively. The low specificity of histology indicates that the pathologists found more positive results when the other tests were negative, which implies that the experience and skills of pathologists are important factors affecting the sensitivity and specificity of the histological test.

Advantages and Limitations of RUT

RUT is a simple, quick, and inexpensive test that is commonly used in medical practice, especially in resource-limited settings [5,23]. It also does not necessitate the participation of a pathologist. Furthermore, Uotani and Graham mentioned that "RUT can be used as an informal assessment of the pathology laboratories' accuracy. If there is a discrepancy between RUT and histology results, especially a positive RUT and negative histology, the histopathology should be reviewed and discussions with the pathologist should be held" [5].

To be positive for RUT, the biopsy sample must contain at least 10^5 ^*H. pylori *[5,23,24]. Hence, anything that reduces bacteria concentration, such as the recent use of antibiotics and proton pump inhibitors (PPI), intestinal metaplasia, and incorrect biopsy sampling, can result in a false negative result [4,22,23]. Stopping PPI before two weeks of the test is advised to reduce the possibility of false negativity. RUT results are also time-sensitive. The higher the concentration of bacteria and the warmer the temperature, the faster the color change [5,13,22]. Most positive results are documented within two to three hours, but it is recommended to wait 24 hours before declaring a negative test. In addition, there is a risk of false positive RUT if other urease-producing bacteria are present in the sample, especially if the contact time exceeds 24 hours [13,25].

Special Situations and Which Test to Choose?

Diagnosis in Pediatrics Population

Several studies have found that the density and distribution of *H. pylori* in the pediatric age group are low, particularly in children under the age of five years, increasing the possibility of false negative results. Therefore, it is suggested that histology and RUT be used in children to identify *H. pylori* [19].

Diagnosis in Peptic Ulcer Bleeding (PUB) Patients 

Several prior reports have shown that in PUB, the sensitivity of both invasive tests is low. One of the causes is the recent use of PPI for bleeding, which reduces the number and density of bacteria [4,20,22,26]. Another reason is intragastric contact between blood proteins containing killing factors and bacteria [20]. Though histology's sensitivity decreases with bleeding, it remains the most reliable invasive test. Multiple studies have also revealed higher histological sensitivities with the use of modified Giemsa stain [17].

Diagnosis in Patients With Atrophic Gastritis (AG) and Intestinal Metaplasia (IM)

The great majority of biopsies test negative for the organism when there is atrophic change and metaplasia, resulting in hypochlorhydria and an unfavorable environment [13,17,22]. AG and IM start in the antrum and expand to the corpus along the lesser curvature after chronic *H. pylori* infection. At the same time, bacteria colonization is shifting to the proximal stomach, corpus, and fundus because of unfavorable antral conditions such as high PH. Therefore, the lesser curvature including the antrum is not a good biopsy site in these conditions [17].

The sensitivity of RUT decreases significantly in the above-mentioned two conditions. This is because AG and IM create a less acidic environment, which allows the bacteria to produce less urease, increasing the possibility of false negativity. As a result, RUT will have a lower sensitivity. Histology with Giemsa stain, on the other hand, is not affected as the test is not dependent on urease activity [17]. In summary, the histopathologic method with biopsy of greater curvature and proximal stomach provides the most sensitivity and specificity in patients with AG and IM [17].

Limitations

This systematic review has several limitations. We only included articles written in English and published in the past 10 years. Also, studies whose free full texts were not accessible were not reviewed. The studies that were not included could have provided additional relevant information, which would have enhanced the quality of the review.

## Conclusions

Both histopathologic tests and RUT have a high and comparable sensitivity and specificity in dyspeptic patients. However, they vary in different clinical situations, making one test a better choice than the other. They both have limitations and either of them cannot be used as a single gold standard test. In summary, in patients with PUB, AG, and IM, histology with modified Giemsa stain is a better choice. It is also superior to RUT in patients taking PPI. Though noninvasive tests like stool antigen and breath tests are used to ensure eradication, among invasive tests, histology is a better choice in the follow-up of eradication therapy. RUT, on the other hand, is a better choice compared to histology in a resource-limited setting and if the results are needed faster.

Therefore, to maintain high sensitivity and specificity and accurately diagnose the pathogen, it is important to use at least two diagnostic tests depending on the clinical situation. In addition, using the Sydney system recommendation for biopsy numbers and sites elevates the diagnostic performance of the tests. We also recommend that more studies be conducted on diagnostic tests of *H. pylori* to find a reliable gold standard test.
